# Long non-coding RNA MALAT1 regulates oxaliplatin-resistance via miR-324-3p/ADAM17 axis in colorectal cancer cells

**DOI:** 10.1186/s12935-020-01549-5

**Published:** 2020-09-29

**Authors:** Changru Fan, Qiulan Yuan, Guifeng Liu, Yuliang Zhang, Maojun Yan, Qingxu Sun, Chaoyu Zhu

**Affiliations:** Department of Abdominal Surgery, Linyi Cancer Hospital, No. 6 Lingyuan East Road, Linyi, 276001 Shandong China

**Keywords:** MALAT1, miR-324-3p, ADAM17, Ox-sensitivity, CRC

## Abstract

**Background:**

Colorectal cancer (CRC) is one of the most general malignant tumors. Accumulating evidence implied that long non-coding RNA Metastasis Associated Lung Adenocarcinoma Transcript 1 (MALAT1) participated in the tumorigenesis of CRC. However, the effect of MALAT1 in drug-resistance needed to be further illustrated.

**Methods:**

Levels of MALAT1, microRNA (miR)-324-3p, and a disintegrin and metalloprotease metallopeptidase domain 17 (ADAM17) were detected using quantitative real-time polymerase chain reaction (qRT-PCR) or western blot assay. Cell Counting Kit 8 (CCK-8) was used to assess the half maximal inhibitory concentration (IC50) of oxaliplatin (Ox). Meanwhile, cell proliferation, migration and apoptosis were detected by CCK-8, transwell assay, and flow cytometry, respectively. The interaction between miR-324-3p and MALAT1 or ADAM17 was clarified by dual-luciferase reporter assay. Also, the effect of MALAT1 on tumor growth was detected in xenograft tumor mice treated with Ox.

**Results:**

Significant up regulation of MALAT1 and ADAM17, and decrease of miR-324-3p were observed in Ox-resistant CRC tissues and cells. MALAT1 deficiency enhanced the sensitivity of Ox-resistant CRC cells response to Ox, while miR-324-3p repression or ADAM17 acceleration could overturn this effect. Moreover, MALAT1 silencing repressed tumor growth in Ox-treated nude mice. Mechanically, MALAT1 exerted promotion effect on the resistance response to Ox via miR-324-3p/ADAM17 axis in Ox-resistant CRC cells.

**Conclusion:**

MALAT1 modulated the sensitivity of Ox through ADAM17 in Ox-resistant CRC cells by sponging miR-324-3p, thus MALAT1 might serve as a novel insight for the therapy of CRC.

## Highlights

1. MALAT1 and ADAM17 were highly expressed, while miR-324-3p was down regulated in Ox-resistant CRC tissues and cells.
2. Knockdown of MALAT1 could boost the sensitivity of Ox-resistant CRC cells response to Ox.
3. Either miR-324-3p inhibition or ADAM17 increase could abolish the effect of MALAT1 deletion on Ox-sensitivity in vitro.
4. MALAT1 regulated the tumorigenesis via miR-324-3p/ADAM17 axis in Ox-treated mice.

## Background

Colorectal cancer (CRC) is one of the leading causes which contributed to cancer-related deaths, and more than 1.2 million new cases of CRC are diagnosed every year all over the world [1, 2]. Recently, oxaliplatin (Ox)-based chemotherapy was identified as one of the most common therapeutic strategies after surgical resection [3]. Ox combines with 5-fluorouracil and leucovorin (FOLFOX), and this combination can promote the drug-response rate (over than 50%) and improve the median survival for the metastatic CRC [4]. However, the outcomes of metastasis and chemoresistance after receiving chemotherapy are the master barrier to the efficiency of CRC therapy [5]. Investigating the potential mechanism and finding therapeutic biomarkers are necessary to develop the drug-therapies for CRC patients.

Long non-coding RNAs (lncRNAs) with over than 200 nucleotides can exert the functional effects by modifying and interacting numerous types of genes and proteins through mountainous mechanisms [6, 7], flowed by participating in the fundamental pathogenesis, such as carcinogenesis and cardiovascular diseases [8–10]. The function of lncRNAs in cancer progression attracted extensive attentions over the past decades, numerous lncRNAs were identified to be implicated in carcinogenesis by working as oncogenic or tumor suppressor genes [11]. Metastasis-associated lung adenocarcinoma transcript 1 (MALAT1) is a well-known lncRNA, which is strikingly up regulated in liver cancer and cervical cancer [12, 13] and acts vital roles in the development of human cancers and chemoresistance [14, 15]. In CRC, MALAT1 can contribute to the tumor development via releasing oncogene polypyrimidine tract binding protein [16]. Also, MALAT1 has been reported to reduce the Ox-sensitivity, thereby decreasing the therapeutic effect via repressing miR-218 [17]. Thus, it was necessary to completely understand the function of MALAT1 in the Ox-resistance response of CRC cells.

Recently, miRNAs are regarded as a class of non-coding RNAs, and it can partially complement with messenger RNA (mRNA), thereby curbing the expression of targeted proteins [18]. Previously, several miRNAs have been confirmed to be involved in the pathogenesis of human cancers [19]. Emerging researches also suggested that miRNAs were strictly related to chemoresistance in diverse cancers [20]. Moreover, growing number of miRNAs were identified to be associated with the processes of CRC. For instance, miR-324-3p was a key regulator in the radioresistance of nasopharyngeal carcinoma by targeting oncogene WTN2B [21, 22]. In this study, we aimed to explore whether miR-324-3p could account for the Ox-sensitivity in CRC. Furthermore, A disintegrin and metalloprotease (ADAM) metallopeptidase domain 17, a member of ADAMs family, was demonstrated to be related to the drug-resistance in CRC.

In the paper, we focused on the expression of MALAT1, miR-324-3p, and ADAM17 in Ox-resistant CRC tissues and cells. Moreover, we highlighted the potential mechanisms of MALAT1 in modifying the sensitivity of Ox-resistant CRC cells response to Ox.

## Materials and methods

### Clinical specimens and cell treatment

Ox-resistant CRC patients (n = 40) and Ox-sensitive CRC patients (n = 40) were recruited from Linyi Cancer Hospital. And the CRC specimens were received from each participator. Tumor response was verified in accordance with the Response Evaluation Criteria in Solid Tumors (RECIST). Besides, written informed consents were gained from all participators based on the guidelines authorized by the Ethics Committee of Linyi Cancer Hospital.

For cell culture, the CRC cell lines (HCT116 and HCT8) and human fetal normal colonic cells (FHC) were purchased from American Tissue Culture Collection (Manassas, VA, USA). HCT116 and HCT8 were maintained in McCoy’s 5A medium (Hyclone, Logan, UT, USA) and Roswell Park Memorial Institute-1640 (RPMI-1640; Hyclone), respectively. FHC were cultured with dulbecco’s modified eagle medium/F12 (DMEM/F12; Hyclone). All the mediums were supplemented with 10% fetal bovine serum (FBS; Gibco, Carlsbad, CA, USA), penicillin–streptomycin (Gibco; 100 U/mL penicillin and 100 mg/mL streptomycin) at 37 °C in 5% CO_2_ and 95% Moist air.

### Development of Ox-resistant cell lines

This protocol was referenced from the previous descriptions [3]. Briefly, HCT116 and HCT8 cells were cultured in relative media with the addition of 0.1 μmol/L oxaliplatin (Ox; Selleck Chemicals, HOU, USA). The surviving cells were grown and passaged twice (over 9 days) to measure viability. Then, the survival cells were exposed in the complete medium with 0.5 μmol/L (15 days), 1.0 μmol/L (30 days), and finally to 2 μmol/L. The Ox-resistant CRC cell lines were named as HCT116/OxR and HCT8/OxR.

### Cell transfection

Specific short hairpin (shRNA) targeting MALAT1 (sh-MALAT1) and its negative control (sh-NC) were synthesized in RiboBio (Guangzhou, China). The full-length of ADAM17 was inserted into pcDNA3.1 to create overexpression vector (ADAM17), and the scrambled sequence was cloned into pcDNA3.1, thereby generating over expression control (pcDNA). Besides, the miR-324-3p mimic (miR-324-3p), miR-324-3p inhibitor (anti-miR-324-3p) and their controls (miR-NC and anti-miR-NC) were obtained from HANBIO (Shanghai, China). The relative sequences were respectively transfected into HCT116/OxR and HCT8/OxR cells using Lipofectamine 3000 reagent (Invitrogen, Carlsbad, CA, USA). Moreover, sh-MALAT1 or sh-NC with lentiviral vector was used to form Ox-resistant CRC cells stably.

### Quantitative real-time polymerase chain reaction (qRT-PCR) assay

The total RNA from tumor Ox-resistant CRC tissues and cells was extracted with Trizol reagent (Invitrogen). The total RNA was reversely transcribed into complementary DNA (cDNA) utilizing PrimeScript RT reagent Kit (Takara, Dalian, China). Then, qRT-PCR was carried out with the synthesized cDNA, corresponding primers, and the reagent of SYBR Prime Script RT-PCR Kits (Takara). The levels were calculated using the 2^−ΔΔCt^ method. U6 small nuclear 1 (U6, Gene ID: 26827) and glyceraldehyde-3-phosphate dehydrogenase (GAPDH, GeneID: 2597) were used to standardize the expression of miR-324-3p (Gene ID: 442898), MALAT1 (GeneID: 378938) and ADAM17 (GeneID: 6868). The primers were as listed: MALAT1 (Forward: 5′-GGGTGTTTACGTAGACCAGAACC-3′, Reverse: 5′-CTTCCAAAAGCCTTCTGCCTTAG-3′); miR-324-3p (Forward: 5′-ACTGCCCCAGGTGCTGCTGG-3′, Reverse: 5′-TGTCAAAAGGGAAATGAGGC-3′); ADAM17 (Forward: 5′-GTGCAGGGTCCGAGGT-3′, Reverse: 5′-GCGAGCACAGAATTAATACGAC-3′); GAPDH (Forward: 5′-GCACCGTCAAGGCTGAGAAC-3′, Reverse: 5′-ATGGTGGTGAAGACGCCAGT-3′); U6 (Forward: 5′-CTCGCTTCGGCAGCACA-3′, Reverse: 5′-AACGCTTCACGAATTTGCGT-3′).

### Cell Counting Kit 8 (CCK-8)

The cells were seeded in a 96-well plate and cultured for 72 h with different dose of Ox. Then, 10 mg/mL of CCK8 reagent was added, and cell viability was detected on the base of the protocols. The absorbance at 450 nm was measured using a microplate reader. The half-maximal inhibitory concentration (IC50) of Ox was determined in HCT116/OxR and HCT8/OxR cells.

### Transwell assay

For the detection of cell migration in CRC, cells cultured in the serum-free medium were seeded on the upper chamber (Corning Incorporated, Big Flats, NY, USA) pre-coated with Matrigel (BD Biosciences, San Jose, CA, USA). The lower chamber supplemented with DMEM containing 10% FBS was used as a chemoattractant. After incubation for 24 h at 37 °C, the migrated cells were fixed, stained, and counted using a microscope (Nikon Eclipse TS100; Nikon, Tokyo, Japan).

### Flow cytometry assay

HCT116/OxR and HCT8/OxR cells were cultured in McCoy’s 5A or RPMI-1640 contained 10% FBS with 2 μmol/L Ox, respectively. After incubation for 48 h, cells were digested using trypsin (Gibco) and washed using Phosphate Buffer solution (PBS; Hyclone). Then, these cells were incubated with Annexin V-fluorescein isothiocyanate (FITC)/propidium iodide (PI) reagent (Vazyme, Nanjing, China). The apoptotic signals were analyzed using flow cytometer.

### Dual-luciferase reporter assay

StarBase v3.0 was used to predict the binding sites between MALAT1 and miR-324-3p, and the complementary sequences between miR-324-3p and the 3′ Untranslated Regions (3′UTR) of ADAM17 were predicted by Target Scan7.2. The common fragments between miR-324-3p and MALAT1 or ADAM17 were cloned and inserted into pmirGLO (Promega, Madison, WI, USA), forming WT-MALAT1 and ADAM17-WT. Similarly, the relative mutants (MUT-MALAT1 and ADAM17-MUT) were constructed in the same way. The reporters were co-transfected with miR-324-3p or miR-NC, respectively., Finally, the luciferase activity was evaluated through adopting a Dual-Luciferase Reporter Assay System kit (Promega).

### Western blot assay

Western blot analysis was performed as previously described [23]. Briefly, protein was extracted and loaded on a 10% polyacrylamide gel. Then, proteins were transferred onto Polyvinylidene Fluoride (PVDF; Millipore, Bedford, MA, USA), and incubated with primary antibody including ADAM17 (ab2051, 1:1000, Abcam, Cambridge, MA, USA), Bcl-2 (ab182858, 1:2000, Abcam), Cleaved caspase-3 (ab2302, 1:1000, Abcam), E-cadherin (14472, 1:1000, Cell signaling technology, Boston, MA, USA), Vimentin (5741, 1:1000, 5741, Cell signaling technology) and GAPDH (ab8245, 1:6000, Abcam) overnight at 4 °C. GAPDH was used as a internal control. Then, the membranes were incubated with secondary antibody (Abcam) for 40 min at temperature, and an enhanced chemiluminescent substrate kit (Millipore) was employed to visualize the unique proteins.

### Xenograft tumor model

The male nude BALB/c mice (n = 6/group) at the age of 6 weeks with 16–20 g weight were purchased from Vital River Laboratory Animal Technology (Beijing, China). This experiment was performed according to the guidelines of the National Animal Care and Ethics Institution and approved by the Animal Research Committee of Linyi Cancer Hospital. Firstly, the mice were randomly divided into three groups, and HCT116/OxR cells (lentivirus-mediated sh-MALAT1 or sh-NC) with 200 µL PBS were subcutaneously injected into the left flank of the mice. Ox (5 mg/kg) was administered two times every week with intragastric injection. After injection for 1 week, the tumor length and width were measured and recorded every 4 days for total 6 times. The volume was calculated according to the formula: volume = length × width^2^ × 0.5. All mice were sacrificed, the xenograft tumors were fetched and weighed.

### Statistical analysis

Data were exhibited as the mean ± standard deviation (SD). Statistical analysis was performed using Student’s *t*-test (two-group) and one-way analysis of variance (three or more groups) followed by turkey’s test. *P *< 0.05 was deemed statistically significant.

## Results

### MALAT1 was up regulated in Ox-resistant CRC tissues and cells

**Fig. 1:**
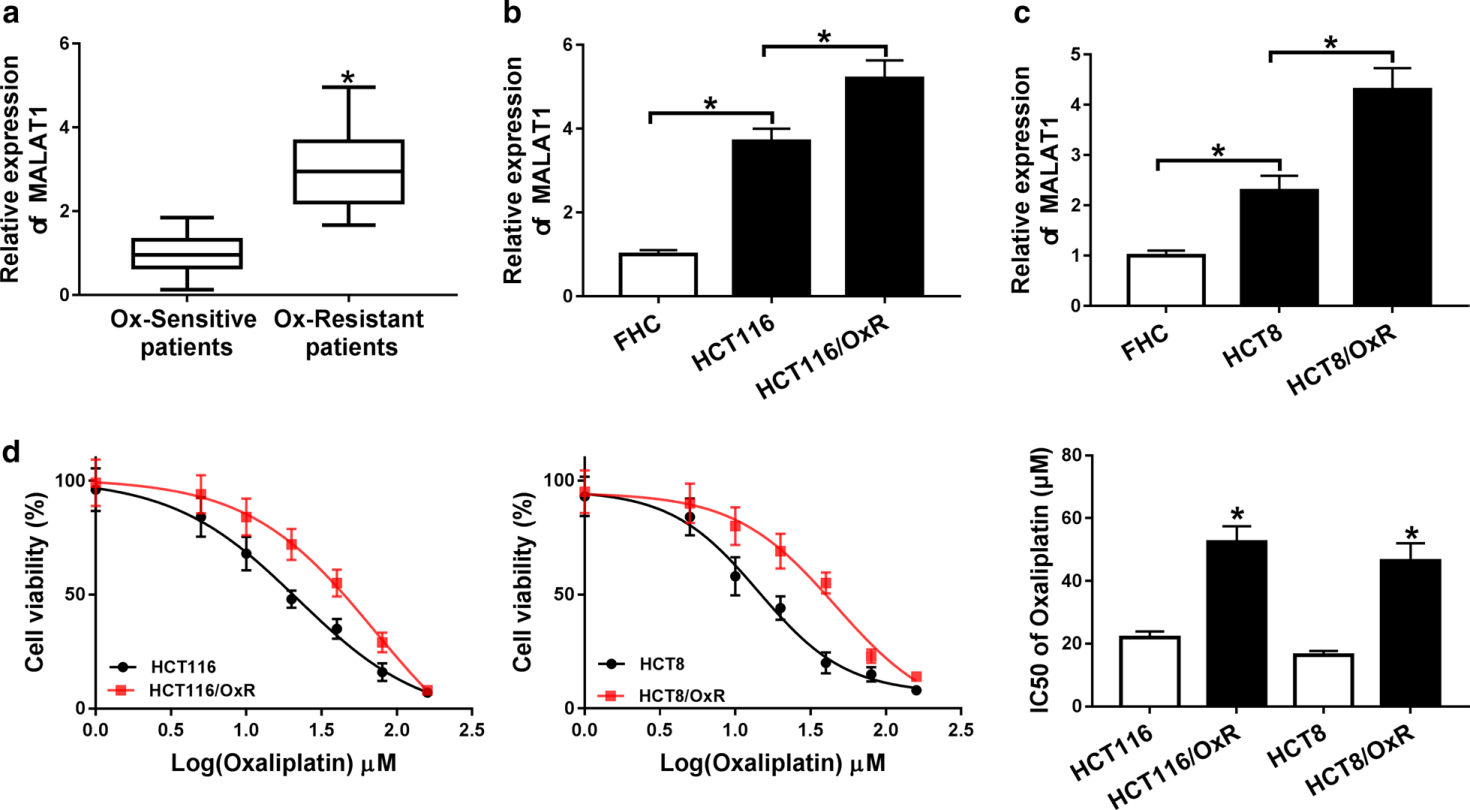
The level of MALAT1 was up regulated in Ox-resistant CRC tissues and cells. **a** QRT-PCR analysis for the level of MALAT1 in Ox-resistant CRC tissues (n = 40) and Ox-sensitive CRC tissues (n = 40). **b**, **c** The expression of MALAT1 in Ox-resistant CRC (HCT116/OxR and HCT8/OxR), CRC (HCT116 and HCT8) and normal cells (FHC). **c** The IC50 value of Ox in HCT116, HCT116/OxR, HCT8 and HCT8/OxR was detected by CCK-8 assay. *P < 0.05

This study recruited Ox-resistant CRC patients (n = 40) and Ox-sensitive CRC patients (n = 40) from Linyi Cancer Hospital and the detailed information of these patients was shown in Table 1. To explore the role of MALAT1 in CRC, we detected the expression of MALAT1 in Ox-resistant CRC tissues and cells. As described in Fig. 1a–c, MALAT1 was highly expressed in Ox-resistant CRC tissues (Fig. 1a) and cells (Fig. 1b, c). Subsequently, we determined the IC50 values of oxaliplatin in both CRC and Ox-resistant CRC cells and found that the IC50 values of oxaliplatin was significantly increased in Ox-resistant CRC cells compared with CRC cells (Fig. 1d). These data indicated that MALAT1 played a crucial role in Ox-resistant CRC.

**Table 1:** Demographic characteristics and clinicopathologic features of colorectal cancer patients (n = 80)

Parameter	Case	Colorectal cancer patients		*P* value
		Ox-resistant (n = 40)	Ox-sensitive (n = 40)	
Age (years)				
≤ 65	42	23	19	0.5021
> 65	38	17	21	
Sex				
Male	41	18	23	0.3711
Female	39	22	17	
Lymphatic metastasis				
No	38	13	25	0.0133*
Yes	42	27	15	
TNM Stage				
I + II	43	17	26	0.0722
III	37	23	14	
Tumor general type				0.4614
Ulcerative	21	14	7	
Mass	48	26	22	
Infiltrative	11	6	7	
Expression of MALAT1				< 0.0001***
Lower	40	2	38	
Higher	40	38	2	

*P < 0.05, ***P < 0.001

### Knockdown of MALAT1 could augment Ox-sensitivity in HCT116/OxR and HCT8/OxR cells

**Fig. 2:**
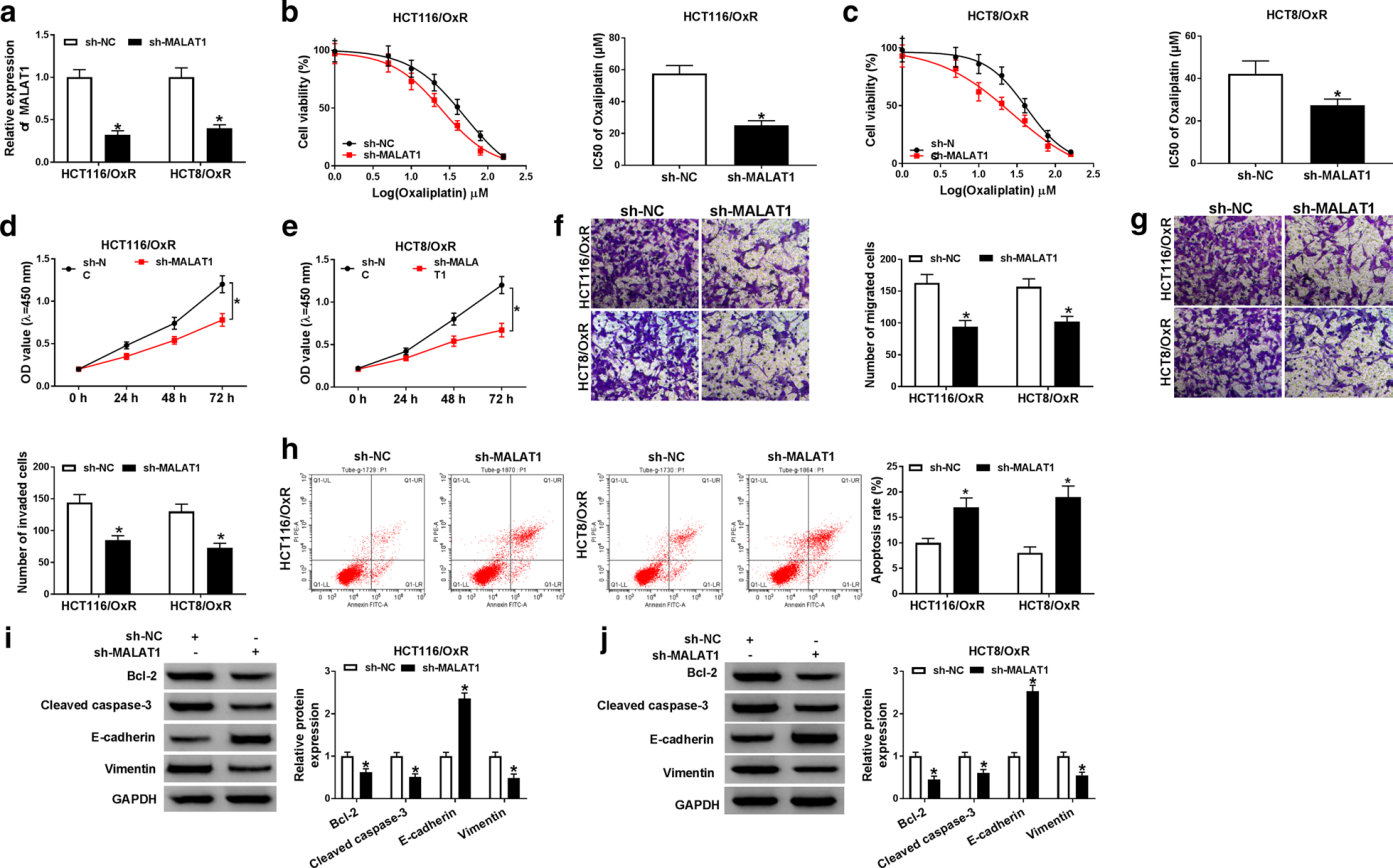
Knockdown of MALAT1 could augment the sensitivity response to Ox in HCT116/OxR and HCT8/OxR cells. **a**–**j** HCT116/OxR and HCT8/OxR cells were transfected with sh-NC or sh-MALAT1. **a** The knockdown efficiency of sh-MALAT1 in HCT116/OxR and HCT8/OxR cells. **b**, **c** The detection of IC50 value of Ox in HCT116/OxR and HCT8/OxR cells. **d**, **e** Measurement of cell viability by CCK-8 assay. **f**, **g** Measurement of cell migration HCT116/OxR and HCT8/OxR cells by transwell assay. **h** Determination of cell apoptosis in HCT116/OxR and HCT8/OxR cells by flow cytometry. **i**, **j** The expression of Bcl-2, Cleaved caspase-3, E-cadherin and Vimentin was detected by western blot. N = 3, *P < 0.05

Firstly, we determined the transfection efficiency of sh-MALAT1 and found the high knockdown efficiency of sh-MALAT1 (Fig. 2a). CCK8 assay manifested that MALAT1 deletion repressed the IC50 values of oxaliplatin (Fig. 2b) and cell proliferation (Fig. 2c) in both HCT116/OxR and HCT8/OxR cells. Moreover, a distinct decrease of cell migration was observed in MALAT1-silenced HCT116/OxR and HCT8/OxR cells (Fig. 2f, g). Also, we examined the effect of MALAT1 silencing on cell apoptosis, flow cytometry indicated that MALAT1 deletion potently triggered cell apoptosis in HCT116/OxR and HCT8/OxR cells (Fig. 2h). Meanwhile, we detected the expression of apoptosis related protein Bcl-2 and Cleaved caspase-3, as well as epithelial to mesenchymal transition (EMT)-related proteins E-cadherin and Vimentin. Our data indicated that MALAT1 down regulation significantly decreased the expression of Bcl-2, Cleaved caspase-3 and Vimentin but increased E-cadherin expression (Fig. 2i and j). Taken together, the deficiency of MALAT1 increased the sensitivity of HCT116 and HCT8 cells to oxaliplatin.

### MiR-324-3p inhibitor overturned the repressive influence of MALAT1 silencing on the Ox-resistance of HCT116/OxR and HCT8/OxR cells

**Fig. 3:**
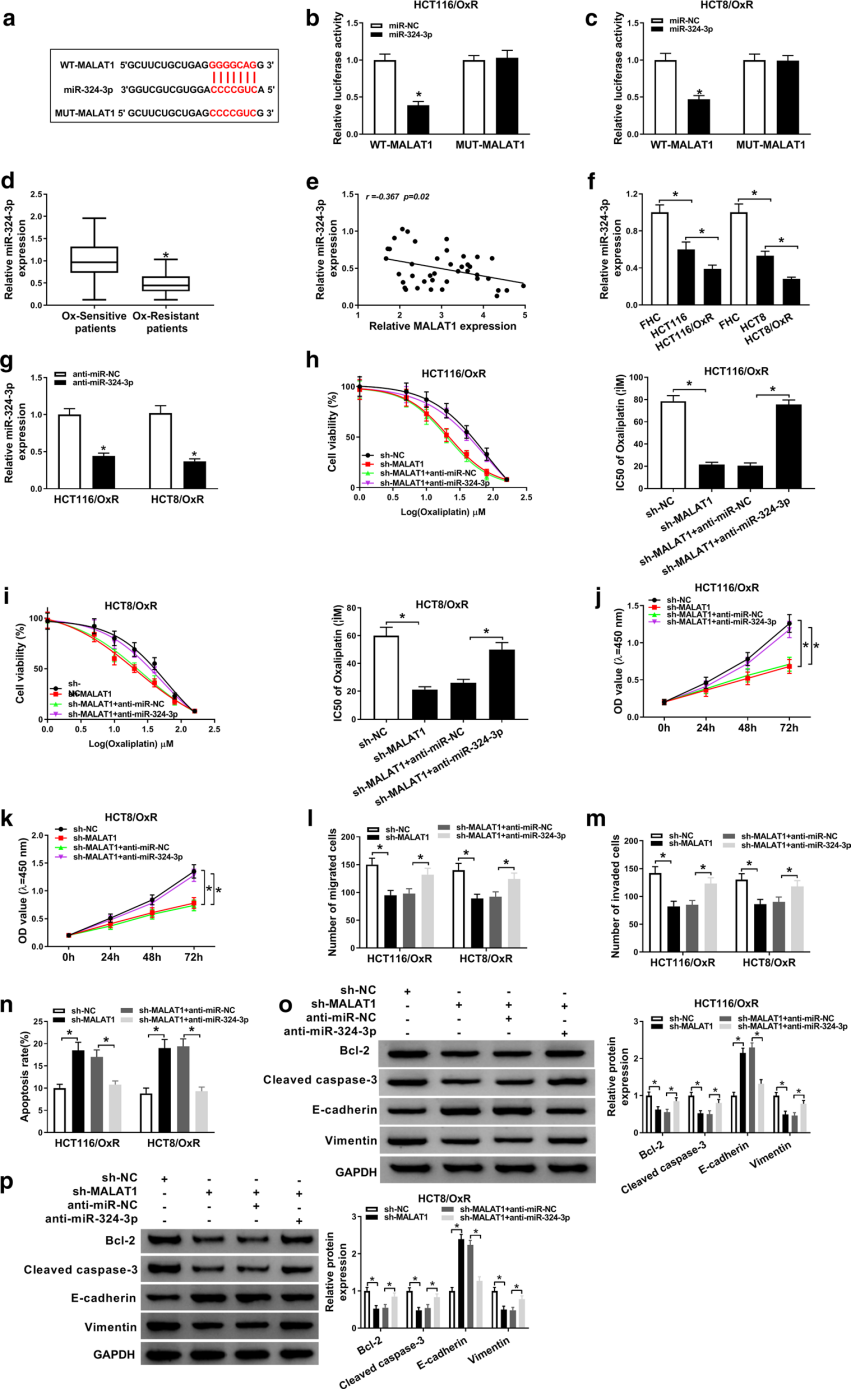
MiR-324-3p inhibitor overturned the effects of MALAT1 silencing on HCT116/OxR and HCT8/OxR cells. **a** The predictive binding sites between MALAT1 and miR-324-3p. **b**, **c** Dual-luciferase reporter analysis for the luciferase activities of WT-MALAT1 and MUT-MALAT1 in Ox-resistant CRC cells. **d** The expression of miR-324-3p in Ox-resistant CRC tissues (n = 40) and Ox-sensitive CRC tissues (n = 40). **e** The relationship between MALAT1 and miR-324-3p in Ox-resistant CRC tissues (n = 40). **f** The level of miR-324-3p in Ox-resistant CRC cells, CRC cells and FHT cells. **g** The transfection efficiency of anti-miR-324-3p in Ox-resistant cells. **h**–**p** HCT116/OxR and HCT8/OxR cells were transfected with sh-NC, sh-MALAT1, sh-MALAT1 + anti-miR-NC, or sh-MALAT1 + anti-miR-324-3p. **h**, **i** The measurement of IC50 value in HCT116/OxR and HCT8/OxR cells response to Ox. **j**, **k** Detection of cell viability by CCK-8 assay. **l**, **m** Measurement of cell migration using transwell assay. **n** Detection of cell apoptosis by flow cytometry. **o**, **p** The expression of Bcl-2, Cleaved caspase-3, E-cadherin and Vimentin was determined by western blot. N = 3, *P < 0.05

The possible miRNAs targets to MALAT1 were predicted by StarBase. There were four miRNAs (miR-5691, miR-1913, miR-324-3p, miR-670-3p) which were predicted to have potential binding sites of MALAT1. Dual-luciferase reporter assay were used to assess the regulatory relationships between these miRNAs and MALAT1. As shown in Additional file 1: Fig. S1A, these miRNAs were negatively regulated by MALAT1, and the negative regulatory relationship between MALAT1 and miR-324-3p was more obvious than the others, thus we chose miR-324-3p to investigate further. The complementary binding sequences between miR-324-3p and MALAT1 was shown in Fig. 3a. To confirm the direct interaction between MALAT1 and miR-324-3p, we performed the dual-luciferase reporter assay. The results validated that miR-324-3p could specifically block the fluorescence intensity of WT-MALAT1, whereas the co-transfection with miR-324-3p had no significant impact on the mutant reporter (Fig. 3b and c). The expression of miR-324-3p was decreased in Ox-resistant CRC tissues (Fig. 3d). In addition, an inverse relationship between MALAT1 and miR-324-3p was determined in Ox-resistant CRC tissues (Fig. 3e). Similarly, we found that miR-324-3p was also down regulated in Ox-resistant CRC cells compared with CRC cells (Fig. 3f). Next, the efficiency of anti-miR-324-3p was verified using qRT-PCR, the level of miR-324-3p was reduced by anti-miR-324-3p (Fig. 3g). Besides, our data suggested that the decrease of IC50 induced by MALAT1 deletion was reversed by miR-324-3p inhibitor in Ox-resistant CRC cells (Fig. 3h, i). Moreover, cell proliferation (Fig. 3j, k) and migration (Fig. 3l, m) inhibited by MALAT1 down regulation were blocked by miR-324-3p deletion in Ox-resistant CRC cells (Fig. 3j–m). We also determined cell apoptosis and discovered that MALAT1 deletion induced cell apoptosis was harbored by miR-324-3p deletion (Fig. 3n). Furthermore, we found that the regulatory effects of MALAT1 deletion on the expression of Bcl-2, Cleaved caspase-3, E-cadherin and Vimentin were reversed by miR-324-3p down regulation (Fig. 3o, p). These data meant that MALAT1 exerted regulatory effects on cell proliferation, migration and apoptosis in Ox-resistant CRC cells via sponging miR-324-3p.

### ADAM17 was a target of miR-324-3p

**Fig. 4:**
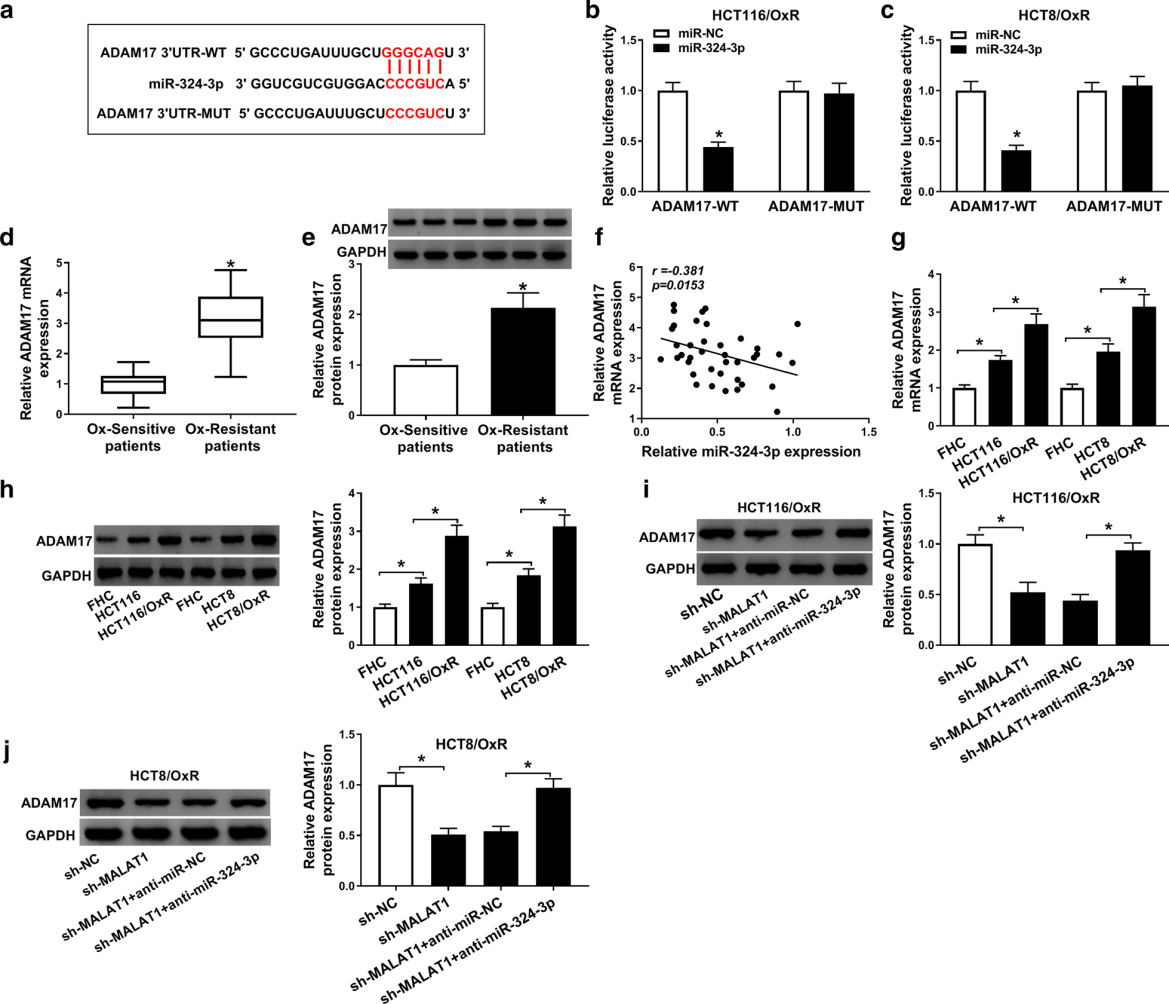
ADAM17 was a target of miR-324-3p. **a** The complementary sequences between miR-324-3p and ADAM17. **b**, **c** The fluorescence intensities of ADAM17-WT and ADAM17-MUT in HCT116/OxR and HCT8/OxR cells transfected with miR-324-3p. **d**, **e** Measurement of ADAM17 mRNA and protein expression in Ox-resistant CRC tissues (n = 40) and Ox-sensitive CRC tissues (n = 40). **f** The relationship between miR-324-3p and ADAM17 in Ox-resistant CRC tissues (n = 40). **g**, **h** Detection of ADAM17 mRNA and protein expression in Ox-resistant CRC cells, CRC cells, and FHC cells. **i**, **j** The protein expression of ADAM17 in HCT116/OxR and HCT8/OxR cells transfected with sh-NC, sh-MALAT1, sh-MALAT1 + miR-NC, or sh-MALAT1 + miR-324-3p. N = 3, *P < 0.05

As predicted by TargetScan7.2, there were binding sites between miR-324-3p and ADAM17 (Fig. 4a). There were three genes (ADAM12, ADAM17 and ADAM22) that probably had the existence of binding sites. Dual-luciferase reporter assay were used to assess the regulatory relationships between these genes and miR-324-3p. Then we tested the regulatory relationships between these genes and miR-324-3p, and the results showed that ADAM17 showed more obvious negative regulatory relationship with miR-324-3p. Subsequently, the relationship between miR-324-3p and ADAM17 was illustrated using dual-luciferase reporter assay, the results presented that the luciferase activity of ADAM17-WT was decreased in HCT116/OxR and HCT8/OxR cells co-transfected with miR-324-3p, but miR-324-3p could not alter the luciferase activity of ADAM17-MUT in vitro (Fig. 4b and c). Besides, the mRNA and protein levels of ADAM17 were up regulated in Ox-resistant CRC tissues (Fig. 4d and e). Moreover, we observed an inverse relationship between miR-324-3p and ADAM17 in Ox-resistant CRC tissues (Fig. 4f). Similarly, we also observed the higher mRNA and protein expression of ADAM17 in Ox-resistant CRC cells in comparison to CRC cells (Fig. 4g, h). As shown in Fig. 4i, j, MALAT1 deletion inhibited the expression of ADAM17, which was reversed by miR-324-3p inhibitor. These results suggested that miR-324-3p was a direct target of ADAM17.

### ADAM17 up regulation could abrogate the effect of MALAT1 reduction on the Ox-sensitivity in CRC cells

**Fig. 5:**
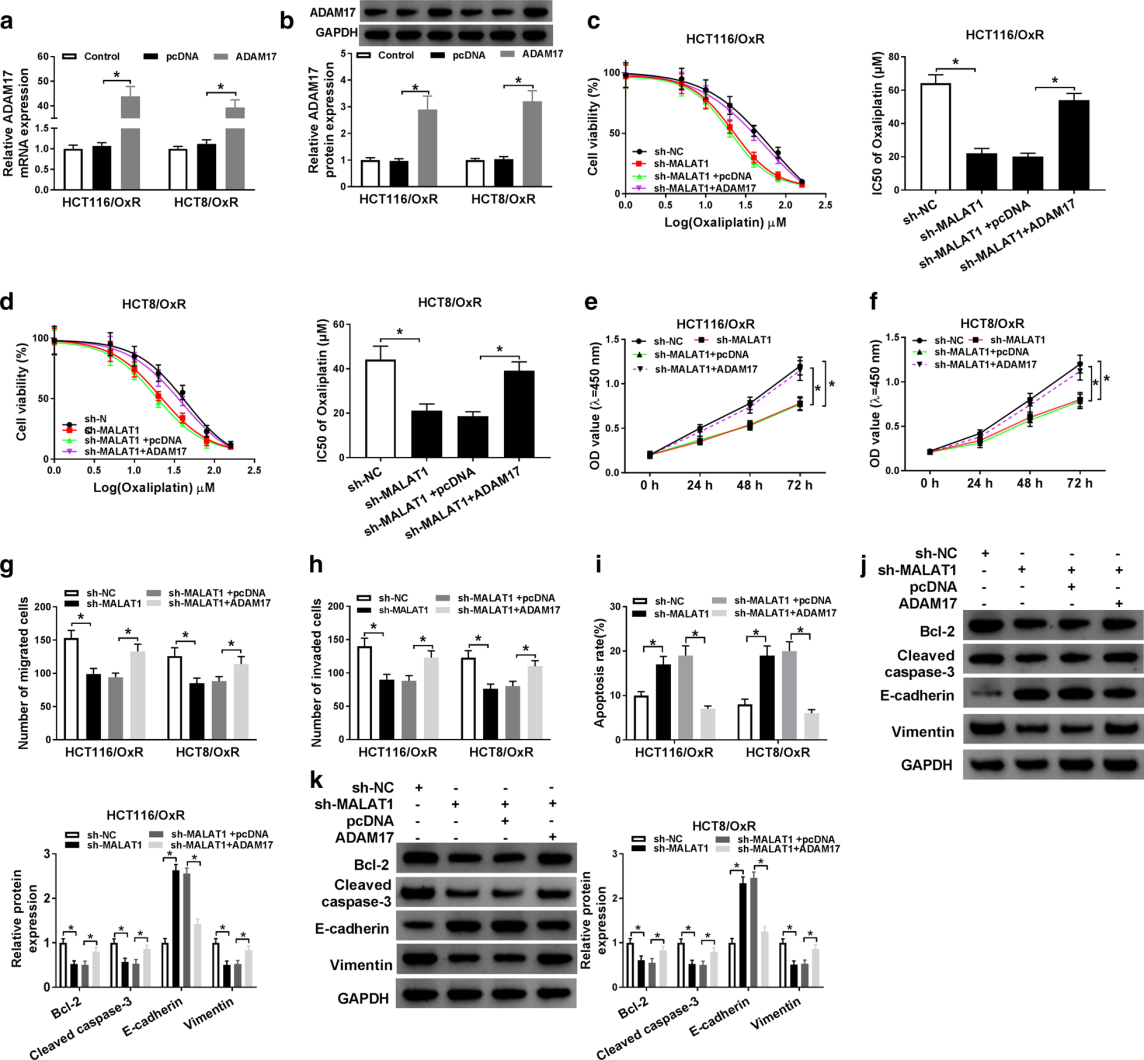
ADAM17 up regulation could abrogate the effect of MALAT1 reduction on the sensitivity of Ox-resistant CRC cells. **a**, **b** The transfection efficiency of ADAM17 in Ox-resistant CRC cells. **c**–**k** HCT116/OxR and HCT8/OxR cells were transfected with sh-NC, sh-MALAT1, sh-MALAT1 + pcDNA, or sh-MALAT1 + ADAM17. **c**, **d** The detection of IC50 value of Ox in HCT116/OxR and HCT8/OxR cells. **e**, **f** Measurement of cell viability by CCK-8 assay. **g**, **h** Measurement of cell migration HCT116/OxR and HCT8/OxR cells by transwell assay. **i** Determination of cell apoptosis in HCT116/OxR and HCT8/OxR cells by flow cytometry. **j**, **k** The expression of Bcl-2, Cleaved caspase-3, E-cadherin and Vimentin was detected by western blot. N = 3, *P < 0.05

Next, we investigated whether ADAM17 could modulate Ox-resistance in CRC. Firstly, the over expression vector of ADAM17 was constructed and transfected into HCT116/OxR and HCT8/OxR cells, the mRNA and protein level of ADAM17 were remarkably enhanced in vitro (Fig. 5a and b). CCK8 analysis displayed that the inhibition effect of MALAT1 knockdown on the IC50 of Ox was eliminated by ADAM17 over expression in vitro (Fig. 5c, d). Moreover, the repression of MALAT1 deletion on cell proliferation (Fig. 5e, f) and migration (Fig. 5g, h) was restored by ADAM17 over expression in HCT116/OxR and HCT8/OxR cells. And the promotion of MALAT1 deletion on cell apoptosis was blocked by ADAM17 over expression in HCT116/OxR and HCT8/OxR cells (Fig. 5i). Furthermore, the inhibition on Bcl-2, Cleaved caspase-3 expression and Vimentin and promotion on E-cadherin expression induced by MALAT1 deletion were reversed by ADAM17 over expression (Fig. 5j, k). All the findings revealed that the effect of MALAT1 deletion on Ox-resistance was rescued by ADAM17 over expression in HCT116/OxR and HCT8/OxR cells.

### MALAT1 silencing promoted Ox-sensitivity in xenograft tumor model

**Fig. 6:**
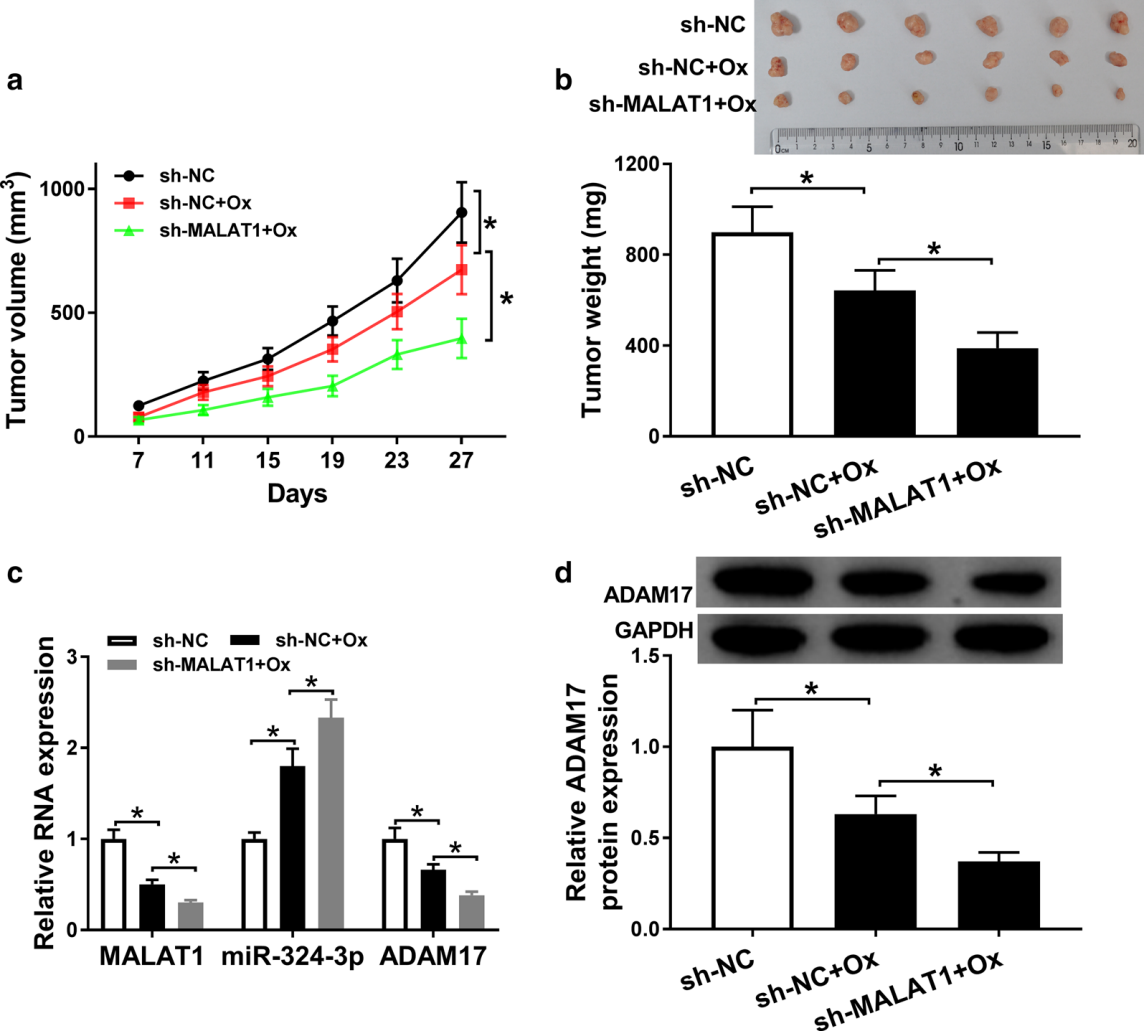
MALAT1 silencing suppressed tumor growth in Ox-resistant xenograft model mice. Sh-MALAT1 or sh-NC was injected into Ox-treated nude mice. **a**, **b** The data of the tumour image, tumor volume and weight under MALAT1 repression. **c** qRT-PCR analysis for the effect of MALAT1 silencing on the levels of MALAT1, miR-324-3p, and ADAM17 in tumors. **d** The protein level of mature ADAM17 in xenograft tumors. N = 3, *P < 0.05

Then, we explored the effects of MALAT1 on tumorigenesis in vivo. After injection for 27 days, the tumor volume and weight were declined by MALAT1 down regulation in Ox-treated mice in comparison with the control (Fig. 6a, b). Moreover, we found that decreased MALAT1 and ADAM17 expression and increased miR-324-3p expression were found in tumor tissues isolated from sh-MALAT1 mice treated with Ox (Fig. 6c). Also, the protein level of ADAM17 was constrained by MALAT1 down regulation in Ox-treated mice (Fig. 6d). Overall, MALAT1 deletion could strengthen the sensitivity response to Ox via miR-324-3p/ADAM17 axis in xenograft tumor model.

## Discussion

Despite current chemotherapeutic methods evidently improved the survival of metastatic tumors, nearly all CRC patients finally developed into chemoresistance with cell metastasis [5]. Understanding the regulatory mechanism of chemoresistance in CRC is utterly important to find the therapeutic strategies for CRC [24]. MALAT1 was firstly reported to be involved in the metastatic development in non-small cell lung cancer [25], and the role of MALAT1 in CRC attracted more attentions recently [16, 26]. MALAT1 has been reported to participate in mountainous biological processes, such as development and metastatic outcomes [27]. In a previous study, the high expression of MALAT1 acted as a poor prognostic biomarker in CRC [28]. In this paper, we focused on the function of MALAT1 in Ox-resistance of CRC. The DNA damage responses acted as the pivotal functions in tumorigenesis and chemoresistance, Ox led to cell apoptosis by stimulating DNA damages [29]. Hence, it was necessary to shed light on the Ox-resistant CRC. Firstly, we found that MALAT1 was highly expressed in Ox-resistant CRC tissues and in established Ox-resistant CRC cells. The high expression of MALAT1 might be inversely associated with Ox-resistance. Moreover, knockdown of MALAT1 could notably decline the IC50 of Ox and inhibit cell proliferation, migration, EMT and promote cell apoptosis in Ox-resistant HCT116 and HCT8 cells. Moreover, MALAT1 knockdown suppressed tumor growth in Ox-treated nude mice. After elucidating the clinical value of MALAT1 in CRC, we tried to clarify the underlying molecular of MALAT1 regulating this Ox-sensitivity and unravel the probable mechanisms on the resistant evolution of CRC. Over the past decades, the interrelation between lncRNAs and miRNAs has been widely acknowledged [30]. According to the previous descriptions, starBase v3.0 software was used to predict the potential targets of MALAT1. As predicted by TargetScan7.2, miR-324-3p was found to be a candidate target of MALAT1. A previous research exposed that miR-324-3p functioned as a distinct biological role in human cancers [31]. MiR-324-3p could modulate the progression of cholangiocarcinoma through targeting ATP-binding cassette transporter A1 [32]. Collectively, miR-324-5p acted as a tumor suppressor in mountainous cancers, including CRC. Moreover, miR-324-5p was tightly associated with cisplatin resistance in non-small cell lung cancer cells [33]. Hence, we explored whether miR-324-3p could participate in the resistant response in CRC. Subsequently, low expression of miR-324-3p was observed, which was involved in the resistance of Ox-resistant CRC cells. Furthermore, miR-324-3p inhibitor could regain the effect of MALAT1 silencing on Ox-resistance in vitro. All findings illuminated that miR-324-3p, a target of MALAT1, was implicated in the Ox-sensitivity in CRC.

Previous studies also validated that miRNA could bind to the 3′UTR of mRNA [34]. In this paper, ADAM17 was predicted to be a target of miR-324-3p by TargetScan7.2. ADAM17 was a key mediator of chemoresistance in HCT116 cells, and its promoting effect on tumor growth was also discovered in previous research [35]. The deletion of ADAM17 specially reduced the secretion of EMT-related cytokine in colon cancer [36]. Taken together, ADAM17 was an essential factor in regulating drug-resistance and cell metastasis of human cancer. In this study, ADAM17 could abolish the regulatory effects of MALAT1 deletion on Ox-sensitivity in Ox-resistant HCT116 and HCT8 cells. These findings indicated that ADAM17 functioned as a promotor in Ox-resistance in vitro.

## Conclusion

In summary, high expression levels of MALAT1 and ADAM17, and low level of miR-324-3p were observed in Ox-resistant CRC tissues and cells. MALAT1 deficiency could accelerate Ox-sensitivity in HCT116/OxR and HCT8/OxR cells, while miR-324-5p reduction or ADAM17 up regulation could reverse this effect. More importantly, MALAT1 exerted promotion effect on Ox-resistance via miR-324-3p/ADAM17 axis in vitro and in vivo. MALAT1 might serve as a novel biomarker in Ox-resistant CRC patients. Nevertheless, the further explorations were needed in the further.

## Supplementary information

**Additional file 1: Fig. S1.** MALAT1-miRNAs and miR-324-3p-mRNAs interactions are predicted using StarBase and TargetScan7.2 software. (A) Dual-luciferase reporter was detected to analyze the regulatory relationships between miRNAs and MALAT1. (B) Dual-luciferase reporter was detected to analyze the regulatory relationships between mRNAs and miR-324-3p. N = 3, *P < 0.05, **P < 0.01, ***P < 0.001.

## Data Availability

All data generated or analysed during this study are included in this published article.
