# Supplementary figures and images for: Long non-coding RNA MALAT1 regulates oxaliplatin-resistance via miR-324-3p/ADAM17 axis in colorectal cancer cells
Source: Cancer Cell Int. 2020 Sep 29;20:473. doi: 10.1186/s12935-020-01549-5 (PMC7525982; doi:10.1186/s12935-020-01549-5)

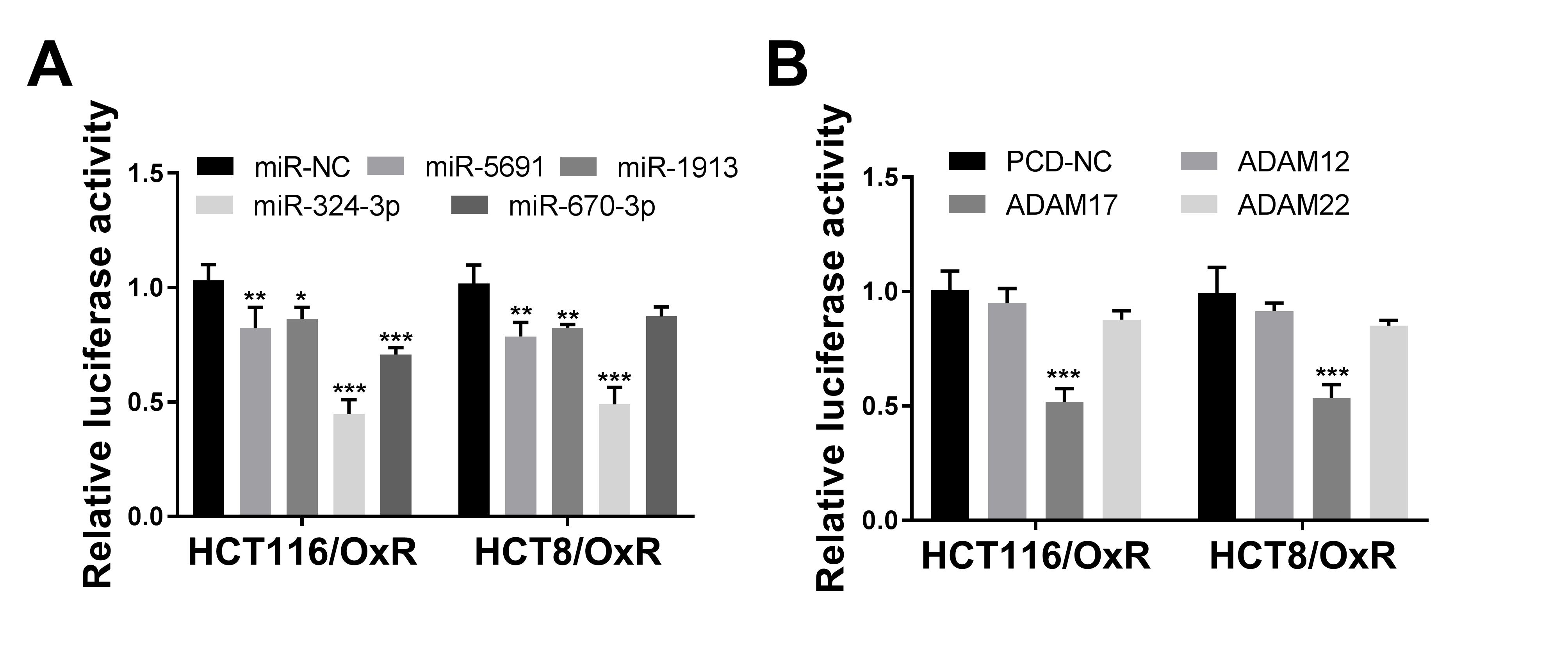

Supplement: Supplementary file 1 — Additional file 1: Fig. S1. MALAT1-miRNAs and miR-324-3p-mRNAs interactions are predicted using StarBase and TargetScan7.2 software. (A) Dual-luciferase reporter was detected to analyze the regulatory relationships between miRNAs and MALAT1. (B) Dual-luciferase reporter was detected to analyze the regulatory relationships between mRNAs and miR-324-3p. N = 3, *P < 0.05, **P < 0.01, ***P < 0.001. [file 12935_2020_1549_MOESM1_ESM.tif]
